# Health Behaviors of Cancer Survivors According to the Employment Status and Occupation: A Cross-Sectional Study

**DOI:** 10.3390/healthcare11222974

**Published:** 2023-11-16

**Authors:** Ka Ryeong Bae, Wi-Young So, Su Jung Lee

**Affiliations:** 1 Samsung Advanced Institute of Health Sciences & Technology (SAIHST), Sungkyunkwan University, Seoul 06355, Republic of Korea; baekr8385@skku.edu; 2 Sport Medicine Major, College of Humanities and Arts, Korea National University of Transportation, Chungju-si 27469, Republic of Korea; wowso@ut.ac.kr; 3 School of Nursing, Research Institute of Nursing Science, Hallym University, Chuncheon 24252, Republic of Korea

**Keywords:** cancer survivors, health behavior, work, employment, occupations

## Abstract

This study aimed to identify differences in health behaviors according to the employment status and occupation of cancer survivors, as well as to identify risk factors. Using data from the Korea National and Health Nutrition Examination Survey (2008–2018), 1023 cancer survivors aged 19–60 years were classified based on their employment status and occupation, and their health behaviors were comparatively assessed. To investigate the impact of occupational status on the health behaviors of cancer survivors, we performed multivariate adjusted logistic regression analysis. Five hundred fifty-six (54.3%) cancer survivors were engaged in economic activities. After adjusting for various factors, white- and blue-collar workers exhibited an increased risk of obesity. The blue-collar group had a 1.45 times higher risk of non-practice with cancer screening, while the white-collar group had a 0.50 times lower risk of non-practice with health screening. The results provide evidence of the need to support cancer survivors in practicing healthy behaviors according to their employment status and occupation. As cancer survivors’ economic activities increase, it is necessary to help them manage their health by predicting any possible health-behavior failures.

## 1. Introduction

The proportion of cancer survivors worldwide is increasing rapidly [1]. In Korea, the cancer survival rate reached 70.7% owing to the development of early cancer screening and treatment technology, and the number of cancer survivors was more than 2.15 million as of 2019 [2]. Extending the survival period, and thus increasing the number of cancer survivors, requires cancer management at both the national and individual levels. Cancer survivors should improve their lifestyle and practice healthy behaviors that control their disease progression or the occurrence of secondary cancer [3]. Furthermore, cancer survivors are at a high risk of worsening their underlying diseases and developing new chronic diseases, or secondary cancer owing to the late effects of treatments such as surgery, hormone therapy, and chemotherapy; therefore, health behavior in daily life is extremely crucial [4]. The American Cancer Society emphasizes the practice of health-related behaviors in life, including smoking cessation, reducing alcohol intake, increasing physical activity, and undertaking a second cancer screening [5], which can reduce the risk of cancer recurrence and later side effects for cancer survivors [6,7]. In Korea, there are no official guidelines for cancer survivors; however, in 2006, the Ministry of Health and Welfare and the National Cancer Center established the National Cancer Prevention Guidelines for practicing healthy lifestyles to prevent cancer [8]. According to a recent study on health behavior among cancer survivors, those who have completed cancer treatment consume alcohol more frequently and in larger quantities than patients still undergoing treatment [9]. They also show a lower intention to undergo secondary cancer screening [10]. Moreover, cancer survivors are more likely to continue smoking after treatment [11] and often find it challenging to maintain lifestyle changes such as quitting smoking and engaging in regular exercise [12].

Differences between socioeconomic classes of the population group affect cancer survivors’ health [13]. Specifically, their employment status and occupation influence their physical and mental health and play a crucial role in shaping their health behavior [14]. Among cancer survivors in Korea, the number of people between the ages of 19 and 50—the prime working-age population—increased significantly, from 43% in 2008 to 63% in 2015 [15]. As a significant proportion of cancer survivors return to work after their diagnosis, the number of working cancer survivors is expected to increase [16]. For cancer survivors, economic activity maintains their and their family’s economic quality of life through income maintenance, serves to maintain their sense of identity, and facilitates their self-actualization [17,18,19]. Prior studies have also reported that socioeconomic factors, such as employment status and education level, affect cancer survivors’ health behavior [20,21]. Therefore, it is important to identify differences in health behavior among cancer survivors according to their employment status and occupation, and to identify risk groups. Accordingly, this study investigated the health behavior of cancer survivors in Korea based on their employment status and occupation through a representative National Health and Nutrition Survey conducted nationwide. Our research results are intended for use as basic data for an educational program to improve cancer survivors’ lifestyle and quality of life.

## 2. Materials and Methods

### 2.1. Study Design

This cross-sectional descriptive study analyzed data from the 4th (2008–2009), 5th (2010–2012), 6th (2013–2015), and 7th (2016–2018) Korea National Health and Nutrition Survey (KNHANES) conducted by the Korea Centers for Disease Control and Prevention. The survey employs a two-stage stratified cluster sampling method to ensure a nationally representative sample of individuals aged ≥1 year. The most recent census data were utilized for selecting sample enumeration districts and households. These selections were stratified based on factors such as the region (e.g., city, province, town), age, sex, residential area, and the education level of the household head. All eligible household members aged ≥1 year were included in the analysis. The 4th survey covered 4600 households across 200 districts, the 5th and 6th surveys encompassed 11,520 households in 192 districts, and the 7th survey included 13,248 households in 192 districts, with a total participation of 112,552 individuals during the 4th to 7th National Health and Nutrition Surveys. Survival periods have recently increased, and many cancer survivors are working despite their advanced age. However, in this study, considering Korea’s retirement age standards [22] and the prime working-age population, the age range was restricted to 19–60 among those diagnosed with cancer, and 1099 people were initially selected for analysis. Additionally, 21 participants who did not provide responses regarding their occupational economic activity and 55 participants who did not undergo anthropometric tests were excluded. Ultimately, data from 1023 participants were analyzed.

### 2.2. Measurements

#### 2.2.1. Demographic and Disease Characteristics

Regarding sociodemographic variables, the cancer survivors’ age, sex, household income, marital status, education level, comorbidity, time since diagnosis, and cancer type were investigated. For household income, the average monthly household income presented in the KNHANES was classified as highest, upper middle, lower middle, and lowest. Marital status was classified into two types: those with a spouse or cohabitant, and those without a spouse, including unmarried, bereaved, or divorced. Education level was classified into four groups: ≤6 years, 7–9 years, 10–12 years, and ≥13 years. Regarding comorbidity, participants who answered affirmatively to the question of whether they had been diagnosed with other diseases, such as hypertension, hyperlipidaemias, diabetes, myocardial infarction, angina, stroke, asthma, and/or renal failure, were classified as having a comorbidity. The age at cancer diagnosis and age at survey were evaluated, and the time since diagnosis was classified into three groups [23]: within 2 years, 3–5 years, and more than 6 years after cancer diagnosis. The types of cancer were classified into stomach, colon, breast, cervix, and other. In the original KNHANES data, seven cancer types, including lung and liver, were classified as the most common cancers in Korea; however, the number of lung and liver cancers in this study was extremely small. Therefore, it was included in the “Other” category.

#### 2.2.2. Employment Status and Occupation

The question on employment status was as follows: “Did you work for more than one hour in the past week to earn money?” Participants who answered “yes” were classified as employed, and those who answered “no” were classified as unemployed. Based on previous studies and the Korean Standard Job Classification [24], managers, professionals, and related workers were classified into the ‘white-collar group’; service workers, sales workers, skilled agricultural, forestry and fishery workers, craft and related trades workers, plant/machine operators and assemblers, and elementary workers were classified into the ‘blue-collar group’; and soldiers were excluded.

#### 2.2.3. Health Behaviors

Health behaviors included body mass index (BMI), exercise, current smoking, heavy drinking, cancer screening, and health screening [25]. BMI, which reflects cachexia or obesity status, was calculated using anthropometric measurements. A BMI of less than 18.5 kg/m^2^ was considered underweight, and a BMI of ≥25 kg/m^2^ was considered obese [26]. From 2008 to 2013, exercise-related variables were measured through the International Physical Activity Questionnaire in the Korea National Health and Nutrition Examination Survey. However, starting from 2014, it was replaced with the Global Physical Activity Questionnaire. We found it challenging to precisely measure high-intensity and moderate-intensity exercises. Therefore, only resistance exercise items that remained unchanged in the exercise-related questionnaire items from 2008 to 2019 were used. The actual number of days was measured by asking, “How many days did you perform push-ups, sit-ups, or lift dumbbells, weights, or iron bars at home or the gym during the last week?” Participants who responded two or more days per week were defined as the exercise group in this study [27]. Current smoking was defined as having smoked at least 100 cigarettes in their lifetime and were currently smoking [28]. Heavy drinking was defined as drinking >7 drinks twice a week for men and 5 drinks twice a week for women by measuring the amount and frequency of drinking on a self-report questionnaire [29]. The performance was determined by asking questions about whether cancer screening and health examinations were performed in the past two years.

### 2.3. Statistical Analysis

To investigate the impact of various sociodemographic and employment status variables among cancer survivors, univariate analysis was conducted on obesity, exercise, current smoking, heavy drinking, non-practice of cancer screening, and non-practice of health screening. From the univariate analysis, variables that showed a *p*-value less than 0.05 were identified, including sex, marital status, education level, household income, and comorbidities. We also included age and time since cancer diagnosis as covariates in the multivariate analysis because they are reported to be associated with health behaviors among cancer survivors [30,31]. The variable selection technique employed for the multivariate analysis was the “input” method [32]. A two-sided *p* < 0.05 in the univariate and adjusted logistic multivariate analyses were considered statistically significant.

## 3. Results

### 3.1. Comparison of Sociodemographic Characteristics According to Employment Status and Occupation

Of the 1023 cancer survivors, 467 (45.7%) were unemployed and 556 (54.3%) were employed. Of the employed cancer survivors, 201 (36.2%) were in the white-collar group and 355 (63.8%) were in the blue-collar group. The unemployed group had a high proportion of females (*p* < 0.001) and the lowest household income (*p* < 0.001). Participants in the blue-collar group were older (*p* < 0.001) and less educated (*p* < 0.001)—with comorbidity (*p =* 0.031), time since diagnosis ≤2 years and ≥6 years (*p* = 0.001)—than those in the unemployed and white-collar groups. However, there was no difference between the three groups in marital status and most types of cancer (*p =* 0.153) (Table 1).

### 3.2. Health Behaviors According to Employment Status and Occupation

Regarding health behaviors, the proportion of cancer survivors who were obese (*p* = 0.004), heavy drinkers (*p* = 0.001), and had non-practice of cancer screening (*p* = 0.003) was higher in the blue-collar group. The non-practice of health screening was highest in the unemployed group (*p* = 0.001). However, there were no differences between the three groups in exercise (*p =* 0.243) and currently smoking (*p =* 0.077) (Table 2).

### 3.3. Odds Ratio of Health Behaviors for Sociodemographic Characteristics among Cancer Survivors

Obesity was associated with employment status and occupation, education level, and comorbidity. The risk of obesity in the blue-collar group was 1.79 times higher than in the unemployed group (OR = 1.79, 95% CI = 1.32–2.42, *p* < 0.001). Higher education levels were linked to a lower risk of obesity, with a 0.62– and 0.60–times lower risk in the groups with 10 to 12 years and 13 or more years of education, respectively, compared to the group with less than 6 years of education (OR = 0.62, 95% CI = 0.42–0.90, *p* = 0.012; OR = 0.60, 95% CI = 0.40–0.90, *p* = 0.013). Having a comorbidity increased the risk of obesity by 2.22 times compared to not having a comorbidity (OR = 2.22, 95% CI = 1.63–2.83, *p* < 0.001). Exercise was related to sex, household income, and education level. The level of exercise was higher in males (OR = 2.92, 95% CI = 2.15–3.96, *p* < 0.001), upper middle (OR = 1.94, 95% CI = 1.15–3.27, *p* = 0.013), and highest income groups (OR = 2.30, 95% CI = 1.38–3.83, *p* = 0.001), as well as those with an education level of 7–9 years (OR = 1.87, 95% CI = 1.06–3.29, *p* = 0.031), 10–12 years (OR = 1.98, 95% CI = 1.23–3.20, *p* = 0.005), and 13 years or more (OR = 2.68, 95% CI = 1.64–4.38, *p* < 0.001).

Current smoking status was associated with employment status and occupation, sex, household income, and marital status. The risk of currently smoking in the blue-collar group was 1.74 times higher than in the unemployed group (OR = 1.57, 95% CI = 1.02–2.96, *p* = 0.042). Males had a higher risk of currently smoking (OR = 6.85, 95% CI = 4.07–11.53, *p* < 0.001). The risk of current smoking decreased with a higher household income. Compared to the group with the lowest household income, the risk was 0.39 times lower in the lower middle (OR = 0.39, 95% CI = 0.19–0.83, *p* = 0.014) and upper middle (OR = 0.39, 95% CI = 0.19–0.78, *p* = 0.008) groups, and 0.42 times lower in the group with the highest household income (OR = 0.42, 95% CI = 0.22–0.81, *p* = 0.010). The risk of current smoking was 7.15 times higher among the unmarried participants compared to those who were married (OR = 7.15, 95% CI = 3.68–13.88, *p* < 0.001). The risk of heavy drinking was higher among blue-collar workers (OR = 2.36, 95% CI = 1.49–3.73, *p*<0.001), males (OR = 5.48, 95% CI = 3.62–8.30, *p*<0.001), and unmarried individuals (OR = 2.21, 95% CI = 1.07–4.55, *p* = 0.032). However, those in the highest household income group showed a lower risk of heavy drinking (OR = 0.49, 95% CI = 0.27–0.90, *p* = 0.022).

Non-practice of cancer screening was related to employment status and occupation, sex, household income, education level, and marital status. The blue-collar group (OR = 1.49, 95% CI = 1.10–2.01, *p* = 0.009), men (OR = 1.99, 95% CI = 1.49–2.68, *p* < 0.001), and the unmarried group had a 6.23 times higher risk of not practicing cancer screening compared to the married group (OR = 6.23, 95% CI = 3.39–11.72, *p* < 0.001). However, those in the upper-middle and highest household income groups (OR = 0.57, 95% CI = 0.38–0.86, *p* = 0.008; OR = 0.36, 95% CI = 0.23–0.54, *p* < 0.001), and those with more than 13 years of education (OR = 0.64, 95% CI = 0.43–0.96, *p* = 0.031) were less likely to not practice cancer screening. Non-practice of health screening was related to employment status and occupation, age, household income, education level, and marital status. The risk in the white-collar group was 0.47 times lower than in the unemployed group (OR = 0.47, 95% CI = 0.32–0.69, *p* < 0.001), and in those aged 50 to 60, the risk was 0.68 times lower (OR = 0.68, 95% CI = 0.52–0.89, *p* = 0.004). Additionally, those with the highest household income (OR = 0.37, 95% CI = 0.24–0.56, *p* < 0.001) and those with an education level of ≥13 years had a lower risk of not practicing health screening (OR = 0.57, 95%, CI = 0.38–0.85, *p* = 0.006). However, unmarried subjects had a higher risk of not practicing health screening (OR = 2.86, 95% CI = 1.61–5.07, *p* < 0.001) (Table 3).

### 3.4. Association of Health Behaviors According to Employment Status and Occupation

After adjusting for age, sex, marital status, education level, household income level, comorbidities, and time since cancer diagnosis, white-collar (OR = 1.80, 95% CI = 1.15–2.77, *p* = 0.008) and blue-collar (OR = 1.68, 95% CI = 1.21–2.35, *p* = 0.002) groups showed an increased risk of obesity. Additionally, the risk of non-practice of cancer screening was 1.45 times higher in the blue-collar group (OR = 1.45, 95% CI = 1.04–2.04, *p =* 0.030), and the risk of non-practice of health screening was 0.50 times lower in the white-collar group (OR = 0.50, 95% CI = 0.32–0.78, *p* = 0.002) (Table 4).

## 4. Discussion

This study confirmed that there were significant differences in the health behaviors of cancer survivors depending on employment status and occupation. This study found that cancer survivors with a higher household income and education level showed better health behaviors. This was similar to the results of a study on Korean adults [33] and cancer survivors [20]. In addition, this was consistent with a study showing that cancer survivors with low education levels engage in a variety of unhealthy behaviors, such as smoking, drinking, and a lack of physical activity [34]. This may be because educational level, income, and occupation are important factors, and are related to the ability to understand and acquire health information. In addition, the odds ratio of negative health behaviors, such as current smoking, heavy drinking, and non-practice of cancer and health screening, were higher for unmarried people than for married people. This is similar to the results of previous studies [35,36,37], because unmarried people receive less social support and have a lower motivation to maintain health out of responsibility toward their loved ones. Among the reasons for spousal non-cohabitation, different characteristics appear to depend on the cause, such as widowhood and divorce [35,38]; hence, additional research is needed to identify other factors affecting cancer survivors.

In this study, unemployed cancer survivors had lower or similar health behaviors than employed people. This is similar to the findings of a study that found that unemployed cancer survivors were less likely to currently drink, drink excessively, or smoke compared to employed people, and had similar physical activity levels [39]. Stress caused by unemployment increases an individual’s vulnerability, causing negative lifestyle habits and worsening health [40,41]. However, a systematic review found that previous studies have reported both increased and decreased smoking, drinking, and physical activity in unemployed populations [42]. Because the relationship between unemployment and health is influenced by various variables such as demographic factors, unemployment period, income, and social support, this study appears to have produced different results [43,44]. Additionally, cancer survivors who work may be more likely to participate in social activities that provide opportunities to drink or smoke. It is necessary to encourage unemployed cancer survivors to maintain positive health behaviors and provide information and education so that they can maintain a healthy life and work.

The blue-collar group of cancer survivors exhibited a higher proportion of obesity and non-practice of cancer screening. This is consistent with previous research showing that the prevalence of obesity increases in blue-collar groups due to automation and lack of time, neglecting exercise [45]. Among the blue-collar group, those who worked shift work were found to have high levels of abdominal obesity and hypertriglyceridemia [46]. Blue-collar groups work irregular shifts compared to other groups, which may be the reason they are more likely to develop or sustain unhealthy lifestyle habits [47]. There is a need to provide systematic nutritional education and counseling to prevent and manage diseases caused by obesity in the blue-collar group. Among them, it will be necessary to recognize and intensively monitor the high risk of obesity among shift workers. Limited access to regular health screening in workplaces and difficulties in securing health rights may contribute to these disparities among blue-collar workers [48,49]. Therefore, workplace and social management are needed to allow blue-collar groups to actively participate in national cancer screening projects for the early detection of secondary cancer or cancer recurrence after treatment.

In the white-collar group, the risk odds of being obese was higher than that of the unemployed, and the risk odds of non-practice of health screening was lower. The white-collar group, which is mostly sedentary, is known to have low levels of activity and low physical activity at work [50]. Furthermore, exposure to various environmental factors such as work stress, irregular eating habits, frequent dinners, and insomnia can lead to life imbalance [51]. It will be necessary to raise awareness among the white-collar group that a lack of physical activity and obesity increase the prevalence of metabolic syndrome, and to develop programs that can encourage healthy behaviors after cancer, considering the work of white-collar workers, who mainly work sedentary jobs. In addition, although psychological factors were not considered in this study, it would be necessary to measure depression and stress to confirm the correlation, reflecting research showing that BMI, abdominal obesity, and dyslipidemia increase as job stress increases [52]. In Korea, national health screening for adults began in 1953 when the Labor Standards Act was enacted, mandating employers to conduct health screening for their workers, and is currently being implemented for all citizens over the age of 19 [53]. White-collar workers often undergo health screening because of their high accessibility through on-site checkups [54]. It is necessary to ensure that the unemployed and blue-collar group cancer survivors, who have relatively low accessibility, can receive health screening through publicity through the mass media.

This study requires careful interpretation, considering several limitations. First, this study fails to consider clinical health data that may have influenced the occupation or health behavior of cancer survivors owing to the limitations in analysis using secondary data. Second, as a cross-sectional study, it is challenging to establish a definitive causal relationship between occupational status and health behaviors. Longitudinal or intervention studies are needed to better understand the causal implications. Third, while this study classified economic activity status into broad categories, such as unemployed, blue-collar workers, and white-collar workers, it did not account for more specific employment types, such as regular or non-regular work, or different work characteristics, such as full-time, part-time, and shift work. These nuanced distinctions could influence health behaviors differently and should be considered in future research to provide a comprehensive understanding. Fourth, it is possible that exercise, smoking, drinking, health screening, and cancer screening were either underestimated or overestimated by examining health behaviors through a self-administered questionnaire. Fifth, the severity of cancer and side effects of treatment are crucial factors that may influence cancer survivors’ economic activities and health behaviors. Understanding the influence of these variables can provide valuable insights into cancer survivors’ successful return to work and economic activity. However, as this study did not include these variables, there are limitations to fully evaluating the association between cancer survivors’ health behaviors and their occupational status. Consequently, to obtain comprehensive and accurate results, future research should include these variables in the study design and data collection. Despite these limitations, this study confirmed the employment status of cancer survivors using nationally representative data, and explored the risk of health-related behaviors according to employment status and occupation. In particular, this study is significant in that it provides basic data that can help cancer survivors adopt a healthy lifestyle and further improve their quality of life by identifying its relationship with health behavior.

## 5. Conclusions

Working is important in the lives of cancer survivors, and it is necessary to live a long life while maintaining health through healthy behaviors. This study showed that the health behaviors of cancer survivors differed based on employment status, with unemployed survivors having less and similar health behaviors than employed survivors. Among employed people, the white-collar and blue-collar groups had high odds of being obese, but there was a difference in that the blue-collar group had high odds of not receiving cancer screening, and the white-collar group had low odds of not receiving health screenings. Therefore, it is necessary to identify cancer survivors who are vulnerable to unhealthy lifestyles according to their employment status and occupation to improve health-related quality of life and make health-promotion intervention programs effective.

## Figures and Tables

**Table 1:** Comparison of sociodemographic characteristics according to employment status and occupation (n = 1023).

Variables	Unemployed (n = 467)	Employed (n = 556)		*p*
		White-Collar (n = 201)	Blue-Collar (n = 355)	
	n (%)	n (%)	n (%)	
Age, year, mean ± SD 19–49 50–60	50.18 ± 8.2 190 (40.7) 277 (59.3)	46.9 ± 8.5 119 (59.2) 82 (40.8)	52.1 ± 6.7 94 (26.5) 261 (73.5)	<0.001 <0.001
Female	402 (86.1)	118 (58.7)	237 (66.8)	<0.001
Household income				<0.001
Lowest	97 (20.9)	6 (3.0)	41 (11.5)	
Lower middle	107 (23.1)	26 (12.9)	94 (26.5)	
Upper middle	120 (25.9)	50 (24.9)	131 (36.9)	
Highest	140 (30.2)	119 (52.9)	89 (25.1)	
Marital status				0.153
Married	440 (94.2)	189 (94.0)	344 (96.9)	
Unmarried	27 (5.8)	12 (6.8)	11 (3.1)	
Educational years				<0.001
≤6 years	94 (20.1)	2 (1.0)	81 (22.8)	
7–9 years	58 (12.4)	7 (3.5)	84 (23.7)	
10–12 years	205 (43.9)	53 (26.4)	145 (40.8)	
≥13 years	110 (23.6)	139 (69.2)	45 (12.7)	
Comorbidity				0.031
Yes	163 (34.9)	62 (30.8)	147 (41.4)	
No	304 (65.1)	139 (69.2)	208 (58.6)	
Time since diagnosis				0.001
≤2 years	141 (16.5)	57 (14.9)	75 (21.2)	
3–5 years	132 (28.4)	47 (23.4)	80 (22.6)	
≥6 years	191 (40.9)	97 (48.3)	199 (56.2)	
Type of cancer				
Stomach	55 (11.8)	25 (12.4)	61 (17.1)	0.072
Colon	28 (6.0)	23 (11.4)	30 (8.4)	0.052
Breast	147 (31.5)	42 (20.9)	72 (20.2)	0.001
Cervix	87 (18.6)	37 (18.4)	68 (19.2)	0.948
Other	160 (34.3)	75 (37.3)	129 (36.5)	0.702

**Table 2:** Health behaviors according to employment status and occupation (n = 1023).

Health Behaviors	Unemployed (n = 467)	Employed (n = 556)		*p*
		White-Collar (n = 201)	Blue-Collar (n = 355)	
	n (%)	n (%)	n (%)	
Body mass index, kg/m^2^				0.004
Underweight (<18.5)	28 (6.0)	10 (5.0)	9 (2.5)	
Normal (18.5–22.9)	224 (48.0)	83 (41.3)	141 (39.7)	
Overweight (23.0–24.9)	104 (22.3)	45 (22.4)	78 (22.0)	
Obese (≥25.0)	111 (23.8)	63 (31.3)	127 (35.8)	
Exercise	107 (22.9)	58 (28.9)	84 (23.7)	0.243
Currently smoking	26 (5.6)	11 (5.5)	33 (9.3)	0.077
Heavy drinking	33 (7.1)	23 (11.4)	54 (15.2)	0.001
Cancer screening				0.003
Yes	341 (73.0)	155 (77.1)	229(64.5)	
No	126 (27.0)	46 (22.9)	126 (35.5)	
Health screening				<0.001
Yes	296 (63.4)	158 (78.6)	241 (67.9)	
No	171 (36.6)	43 (21.4)	114 (32.1)	

**Table 3:** Odds ratio of health behaviors for sociodemographic characteristics among cancer survivors.

Characteristics	Obesity		Exercise		Current Smoking		Heavy Drinking		Non-Practice of Cancer Screening		Non-Practice of Health Screening	
	OR (95%, CI)	*p*	OR (95%, CI)	*p*	OR (95%, CI)	*p*	OR (95%, CI)	*p*	OR (95%, CI)	*p*	OR (95%, CI)	*p*
Employment and occupation status												
Unemployed	1.00 (ref)		1.00 (ref)		1.00 (ref)		1.00 (ref)		1.00 (ref)		1.00 (ref)	
White-collar	1.46 (1.02–2.11)	0.449	1.37 (0.94–1.98)	0.103	0.98 (0.48–2.03)	0.961	1.70 (0.97–2.98)	0.064	0.80 (0.55–1.18)	0.267	0.47 (0.32–0.69)	<0.001
Blue-collar	1.79 (1.32–2.42)	<0.001	1.04 (0.75–1.45)	0.801	1.74 (1.02–2.96)	0.042	2.36 (1.49–3.73)	<0.001	1.49 (1.10–2.01)	0.009	0.82 (0.61–1.10)	0.179
Age												
19–49	1.00 (ref)		1.00 (ref)		1.00 (ref)		1.00 (ref)		1.00 (ref)		1.00 (ref)	
50–60	1.05 (0.80–1.39)	0.718	1.15 (0.85–1.54)	0.364	1.04 (0.63–1.71)	0.884	1.38 (0.91–2.10)	0.131	0.83(0.63–1.09)	0.176	0.68 (0.52–0.89)	0.004
Sex												
Female	1.00 (ref)		1.00 (ref)		1.00 (ref)		1.00 (ref)		1.00 (ref)		1.00 (ref)1	
Male	0.85 (0.63–1.15)	0.292	2.92 (2.15–3.96)	<0.001	6.85 (4.07–11.53)	<0.001	5.48 (3.62–8.30)	<0.001	1.99 (1.49–2.68)	<0.001	1.17 (0.87–1.57)	0.305
Household income												
Lowest	1.00 (ref)		1.00 (ref)		1.00 (ref)		1.00 (ref)		1.00 (ref)		1.00 (ref)	
Lower middle	1.41 (0.89–2.22)	0.196	1.42 (0.82–2.48)	0.212	0.39 (0.19–0.83)	0.014	0.68 (0.37–1.28)	0.232	0.67 (0.44–1.03)	0.069	0.82 (0.54–1.05)	0.358
Upper middle	1.13 (0.73–1.75)	0.595	1.94 (1.15–3.27)	0.013	0.39 (0.19–0.78)	0.008	0.82 (0.46–1.46)	0.811	0.57 (0.38–0.86)	0.008	0.68 (0.45–1.02)	0.061
Highest	0.89 (0.58–1.38)	0.613	2.30 (1.38–3.83)	0.001	0.42 (0.22–0.81)	0.010	0.49 (0.27–0.90)	0.022	0.36 (0.23–0.54)	<0.001	0.37 (0.24–0.56)	<0.001
Education level												
≤6 years	1.00 (ref)		1.00 (ref)		1.00 (ref)		1.00 (ref)		1.00 (ref)		1.00 (ref)	
7–9 years	0.98 (0.62–1.54)	0.928	1.87 (1.06–)3.29	0.031	1.12 (0.52–2.40)	0.771	1.14 (0.58–2.27)	0.703	0.90 (0.57–1.43)	0.668	0.94 (0.60–1.47)	0.780
10–12 years	0.62 (0.42–0.90)	0.012	1.98 (1.23–3.20)	0.005	0.71 (0.37–1.39)	0.322	0.92 (0.52–1.63)	0.767	0.76 (0.52–1.11)	0.153	0.75 (0.52–1.08)	0.119
≥13 years	0.60 (0.40–0.90)	0.013	2.68 (1.64–4.38)	<0.001	0.62 (0.30–1.29)	0.202	1.02 (0.56–1.85)	0.960	0.64 (0.43–0.96)	0.031	0.57 (0.38–0.85)	0.006
Marital status												
Married	1.00 (ref)		1.00 (ref)		1.00 (ref)		1.00 (ref)		1.00 (ref)		1.00 (ref)	
Unmarried	1.34 (0.69–2.60)	0.390	1.50 (0.81–2.76)	0.198	7.15 (3.68–13.88)	<0.001	2.21 (1.07–4.55)	0.032	6.23 (3.39–11.72)	<0.001	2.86 (1.61–5.07)	<0.001
Comorbidity												
No	1.00 (ref)		1.00 (ref)		1.00 (ref)		1.00 (ref)		1.00 (ref)		1.00 (ref)	
Yes	2.22 (1.63–2.83)	<0.001	0.78 (0.58–1.06)	0.111	0.85 (0.51–1.42)	0.528	1.21 (0.80–1.82)	0.359	0.87 (0.61–1.07)	0.139	0.80 (0.61–1.06)	0.117
Time since diagnosis, year												
≤2 years	1.00 (ref)		1.00 (ref)		1.00 (ref)		1.00 (ref)		1.00 (ref)		1.00 (ref)	
3–5 years	1.22 (0.84–1.78)	0.290	0.77 (0.51–1.15)	0.196	1.72 (0.82–3.62)	0.152	1.44 (0.80–2.58)	0.221	1.21 (0.82–1.7)	0.340	1.31 (0.91–1.88)	0.150
≥6 years	1.09 (0.78–1.5)	0.622	0.96 (0.69–1.35)	0.820	1.84 (0.95–3.59)	0.073	1.57 (0.94–2.63)	0.084	1.37 (0.98–1.91)	0.066	1.10 (0.79–1.51)	0.577

**Table 4:** Association of health behaviors according to employment status and occupation.

Health Behaviors	Multivariable AOR (95% CI)	*p*
Obesity		
Unemployed	1.00 (ref)	
White-collar	1.80 (1.15–2.77)	0.008
Blue-collar	1.68 (1.21–2.35)	0.002
Exercise		
Unemployed	1.00 (ref)	
White-collar	0.69 (0.44–1.08)	0.102
Blue-collar	0.78 (0.54–1.12)	0.173
Currently smoking		
Unemployed	1.00 (ref)	
White-collar	0.90 (0.37–2.20)	0.810
Blue-collar	1.29 (0.68–2.42)	0.439
Heavy drinking		
Unemployed	1.00 (ref)	
White-collar	1.26 (0.63–2.50)	0.511
Blue-collar	1.67 (1.00–2.78)	0.050
Non-practice of cancer screening		
Unemployed	1.00 (ref)	
White-collar	0.77 (0.48–1.22)	0.266
Blue-collar	1.45 (1.04–2.04)	0.030
Non-practice of health screening		
Unemployed	1.00 (ref)	
White-collar	0.50 (0.32–0.78)	0.002
Blue-collar	0.77 (0.55–1.06)	0.102

OR odds ratio, CI confidence interval. Multivariable logistic regression was adjusted for age, sex, marital status, education level, household income level, comorbidity, and time since cancer diagnosis.

## Data Availability

No additional data available. The Korea Disease Control and Prevention Agency (KDCA) provides the Korea National Health and Nutrition Examination Survey (KNHANES) only to authorized researchers upon request. Information regarding data availability can be found on the KCDA website (https://knhanes.kdca.go.kr/knhanes, accessed on 1 March 2022).
